# Does Intra-Annular Valve Design Equal Intra-Annular Valve Design? Comparison of Two Transcatheter Aortic Valve Prostheses

**DOI:** 10.3390/jcm14061824

**Published:** 2025-03-08

**Authors:** Clemens Eckel, Fadi Al-Rashid, Sophie Bargon, Judith Schlüter, Dagmar Sötemann, Albrecht Elsässer, Johannes Blumenstein, Helge Möllmann, Christina Grothusen

**Affiliations:** 1 Department of Cardiology, St. Johannes Hospital, 44137 Dortmund, Germany; 2 School IV, Carl von Ossietzky Universität Oldenburg, 26129 Oldenburg, Germany; 3 Department of Cardiology, University Hospital Essen, 45147 Duisburg, Germany; 4 Department of Cardiac and Thoracic Vascular Surgery, University Hospital of Schleswig-Holstein, 24105 Kiel, Germany

**Keywords:** TAVI, TAVR, THV, self-expanding, balloon expandable, Navitor, Sapien Ultra, PPM, small annulus

## Abstract

**Background:** Prosthesis-patient mismatch (PPM) has been demonstrated to affect the outcome of both surgical (SAVR) and transcatheter aortic valve replacement (TAVR) patients. Supra-annular transcatheter valves (SAV) appear to offer a superior solution to intra-annular valves (IAV) in this regard. However, data on the comparison of the intra-annular self-expanding (SE) NAVITOR and the intra-annular balloon-expandable (BE) Sapien 3 Ultra in small annuli are limited. **Methods:** A total of 179 patients with severe native aortic valve stenosis were treated with either the SE NAVITOR (SEV; *n* = 104) or the BE Sapien 3 Ultra (BEV; *n* = 75) between March 2019 and June 2024. We compared the clinical and hemodynamic outcomes of the cohort according to the implanted prostheses. BMI-adjusted PPM was defined in accordance with the VARC-3 recommendations. **Results:** The device success at 30 days was superior in patients treated with the NAVITOR prosthesis (94.2% vs. 80.0%, *p* < 0.001), mainly driven by a higher rate of elevated gradients in the BEV group. The post-procedural mean gradient (8.0 mmHg vs. 13.0 mmHg, *p* < 0.001) as well as the rate of moderate to severe prosthesis patient mismatch (12.2% vs. 33.3%, *p* = 0.002) was higher in BEV recipients while the rate of more than moderate paravalvular leakage (PVL) or Valve in Valve (VinV) due to PVL (1.0% vs. 1.3%, *p* = 1.000) was similar between both groups. Pacemaker implantations were numerically more common after SEV (18.4% vs. 8.5%, *p* = 0.110). There was a trend towards higher thirty-day all-cause mortality among patients treated with SAV (3.8% vs. 1.3%, *p* = 0.401). **Conclusion:** The NAVITOR system may offer a more favorable hemodynamic profile compared to the Sapien 3 Ultra device in patients with small aortic annuli.

## 1. Introduction

By overcoming initial challenges of transcatheter aortic valve replacement (TAVR) related to implantation techniques and deepening the understanding of the possibilities and limitations of TAVR, attention is increasingly shifting to how anatomical and technical factors influence the outcomes of the procedure.

TAVI prostheses are categorized into self-expanding (SE) and balloon-expandable (BE) types. In the past, SE prostheses were considered superior to BE prostheses in terms of hemodynamic performance. However, it is possible that the combination of valve position (intra-annular or supra-annular) and design (SE or BE) is more important in determining prosthesis characteristics than the expansion mechanism alone.

Treatment of aortic stenosis in patients undergoing TAVR with small annuli remains challenging and carries the risk of prosthesis-patient mismatch (PPM) [1,2]. Previous studies in populations with small annuli have mainly highlighted the risk of PPM and negative hemodynamic outcomes due to the narrow anatomy [1,2,3]. The recently published *SMall Annuli Randomized To Evolut or SAPIEN* (SMART) trial showed that a supra-annular self-expanding valve (SEV) is noninferior to an intra-annular (IA) balloon expandable valve (BEV) with beneficial lower mean Doppler-derived gradients [4]. Patients were treated with the Evolut Pro/Pro+/Fx valves (Medtronic, MN, USA) in the supra-annular valve group and with the Sapien 3 Ultra valve (Edwards Lifesciences, CA, USA) in the intra-annular valve (IAV) group.

Among SE prostheses, the NAVITOR (Abbott Inc., IL, USA) is characterized by an IA position of the leaflets [5]. This study investigates whether the IA NAVITOR valve also provides an advantage in a subset of patients with small annuli similar to the results of the SMART trial.

## 2. Methods

From March 2019 to June 2024 a total of 179 patients with severe native aortic valve stenosis and small aortic annulus underwent transfemoral TAVR with either the SE NAVITOR (SEV; *n* = 104) or BE Sapien 3 Ultra (BEV; *n* = 75) prosthesis. Retrospective data analysis was performed. In accordance with the SMART trial, small aortic annulus was defined as ≤430 mm^2^. Baseline characteristics, including comorbidities, risk scores, echocardiography, MDCT, electrocardiography, and laboratory and cardiac catheterization data, were recorded in a dedicated database. Similarly, procedural data and complications were also recorded. This study was conducted according to the Declaration of Helsinki and approved by the Ethics Committee of Westfalen-Lippe (2024-454-f-S; 19 July 2024).

### 2.1. Multidetector Computed Tomography

Multidetector computed tomography (MDCT) was performed using a 64-slice or a 192-slice dual-source scanner (Somatom Definition or Somatom Force, Siemens Healthcare, Forchheim, Germany) following previously established protocols [6]. To evaluate the MDCT datasets, a dedicated analysis software (3mensio, Pie Medical, The Netherlands) was utilized. The aortic valve calcium score (AVCS) was measured according to the Agatston method using non-contrast-enhanced MDCT scans [7]. The calcium density (Ca-density) was defined as AVCS/annular area (AU/cm^2^) [8].

### 2.2. Assessment of PPM and PVL

PPM was defined according to Valvular Academic Research Consortium (VARC)-3 criteria as BMI adapted (iAVA if BMI < 30 kg/m^2^: none >0.85 cm^2^/m^2^; moderate 0.85–0.66 cm^2^/m^2^; severe ≤0.66 cm^2^/m^2^; and iAVA if BMI ≥ 30 kg/m^2^: none >0.70 cm^2^/m^2^; moderate 0.70–0.56 cm^2^/m^2^; severe ≤0.55 cm^2^/m^2^) [9]. PVL was evaluated at the time of discharge using a four-class grading scheme (none/trace, mild, moderate, and severe), following the established guidelines and recommendations [9].

### 2.3. Outcomes of Interest

The primary outcome measure was echocardiographic performance, as described by post-interventional gradients, indicated aortic valve area (iAVA), PVL, and possible prosthesis-patient mismatch (PPM), as previously outlined. Secondary outcome measures included 30-day all-cause mortality, technical success, device success at 30 days and the early safety combined endpoint at 30 days, in accordance with the recent VARC-3 document [9].

### 2.4. Statistical Analysis

The population was divided into two subsets according to the type of implanted valve. Continuous data are presented as the median and interquartile range (IQR). Continuous variables were tested for normal distribution using the Shapiro–Wilk test. The groups were compared using the Mann–Whitney U test, Fisher’s two-tailed exact test, or the chi-square test, as appropriate. For all analyses, a two-sided *p*-value of less than 0.05 was considered to be statistically significant. All analyses were conducted using R (version 1 September 2023 (R Core Team (2021); R: A language and environment for statistical computing. R Foundation for Statistical Computing, Vienna, Austria.

## 3. Results

### 3.1. Baseline Data

The mean age was 82.7 [76.0; 86.0] years and 86.0% were female. Baseline parameters and recorded comorbidities differed in terms of age (SEV, 83.0 vs. BEV, 80.0 years; *p* = 0.001), the creatinine clearance (SEV, 52.5 vs. BEV, 62.0, *p* = 0.006), the mean transvalvular gradients (SEV, 43.0 vs. BEV, 40.0 mmHg, *p* = 0.030) as well as the aortic valve calcification (SEV, 1615 vs. BEV, 2083 AU, *p* = 0.010); See Table 1.

### 3.2. Procedural Data and Outcomes

The technical success (SEV, 98.1 vs. BEV, 93.3%, *p* = 0.132) as well as the device success (SEV, 94.2% vs. BEV, 80.0%, *p* = 0.007) were superior after NAVITOR. The rate of pre- (SEV, 96.2% vs. BEV, 6.7%, *p* < 0.001) and post-dilation (SEV, 29.8% vs. BEV, 10.7%, *p* = 0.004) was lower with the Sapien 3 Ultra.

Bleeding type 3–4 was comparable without difference between the groups (SEV, 5.8% vs. BEV, 6.7%, *p* = 1.000). Major vascular complications occurred more often in Ultra recipients (SEV, 0.0% vs. BEV, 6.7%, *p* = 0.012). Major cardiac structural complications were rare overall (SEV, 1.0% vs. BEV, 2.7%, *p* = 0.573). The rate of disabling stroke (SEV, 2.9% vs. BEV, 1.3%, *p* = 0.641) or acute kidney injury stage 2–4 (SEV, 1.9% vs. BEV, 0.0%, *p* = 0.510) was comparable. Pacemaker implantation due to new conduction system alterations were numerically more often after NAVITOR (SEV, 18.4% vs. BEV, 8.5%, *p* = 0.110). The 30-day all-cause mortality showed a numerically higher rate after NAVITOR (SEV, 3.8% vs. BEV, 1.3%, *p* = 0.401); see Table 2. A detailed analysis of the underlying intrahospital causes of death revealed fatal hemorrhage and coronary artery obstruction as the causes of death after NAVITOR; one patient died due to pneumonic sepsis after Ultra. Two further deaths up to 30 days after the implantation (one each after NAVITOR and Ultra) could not be clarified.

### 3.3. Hemodynamics

Transthoracic echocardiography assessment before discharge showed lower mean trans prosthetic gradient (SEV, 8.0 mmHg vs. BEV, 13.0 mmHg, *p* < 0.001) accompanied with a larger orifice area (SEV, 1.9 cm^2^ vs. BEV, 1.7 cm^2^, *p* < 0.001) after NAVITOR. The rate of elevated gradients ≥ 20 mmHg (SEV, 0.0% vs. BEV, 8.2%, *p* = 0.005) and moderate to severe PPM (SEV, 12.2% vs. BEV, 33.3%, *p* = 0.002) was elevated after ULTRA; see Table 2.

## 4. Discussion

In this study, we retrospectively compared the outcome of patients with severe aortic valve stenosis and small annuli treated with two current intra-annular TAVR systems, the SE NAVITOR and the BE Sapien 3 Ultra. Our main results are as follows: (1) The use of the intra-annular SEV platform led to superior hemodynamic results. (2) While vascular complications occurred more often in patients receiving BEV, thirty-day mortality and stroke rates were numerically more often after SEV. (3) The necessity for a permanent pacemaker implantation was numerically higher in patients who underwent SEV implantation.

The expanding field of indications for TAVR in patients with severe aortic valve stenosis requires a more defined treatment with a tailored, individual approach to each patient [10]. This includes a detailed performance analysis of different TAVR platforms. In principle, the SE NAVITOR offers hemodynamic advantages and is characterized by a low rate of PVL [5,11,12]. The BE Sapien is characterized by low PVL and pacemaker rates [13,14]. Recent data from a large, randomized trial provided evidence for the superior durability of a SAV system in comparison to a IAV device in dedicated anatomy of small annuli [4]. Thus, SA TAVR systems are increasingly recommended to avoid severe PPM in small annuli [15,16]. However, the influence of the general implantation mechanism in this anatomy remains unclear when analyzing these platforms. Therefore, data comparing intra-annular prostheses are needed. Here, our data indicate that the SEV may also result in superior hemodynamic results when compared to a BEV platform even though both devices share an intra-annular valve design.

The hemodynamic parameters analyzed in this study included significantly lower transvalvular gradients and effective orifice areas after NAVITOR implantation. In addition, PPM rates were markedly lower with the use of the SEV. This system consists of a metal frame with large cells facilitating coronary access post TAVI and an inner and outer fabric skirt which may limit paravalvular leakage [5]. Calcification is higher in Sapien recipients than in NAVITOR recipients, but native calcification is only moderately increased in the total population. Therefore, a clinically relevant influence on hemodynamics and, in particular, the PVL rate is unlikely in these data. Indeed, we found only one case of more than mild PVL in both groups. In the CHOICE trial, better hemodynamics with a self-expanding supra-annular platform were accompanied by less moderate or severe structural valve deterioration [17]. In order to improve the hemodynamic performance and durability of the small size IA Sapien 3 Ultra, this device was recently modified by using the RESILIA tissue technology. Previous data from surgical trials as well as results from the OCEAN registry indicate a favorably short-term impact of this modification [18].

A potential limitation of the SE NAVITOR is the increased need for a permanent pacemaker implantation [11,19]. In our analysis, 18% of patients underwent pacemaker implantation after TAVR using the NAVITOR device compared to 8.5% after TAVR with a BEV product. In general, a high implantation height is crucial in order to reduce irrevocable damage of the AV-node but has to be weighed against the risk of device pop-out [20]. With regard to the outcome-relevant long-term effects of right ventricular pacing, low pacing rates should be aimed for to avoid pacing-associated cardiomyopathies [21,22]. To address these aspects, the newly introduced successor of the Navitor device—“Vision”—was equipped with radiopaque markers at the preferred implantation depth of 3 mm. In addition, these markers enable intra-commissural alignment to enhance hemodynamic performance and facilitate future coronary access [23,24].

As mentioned, vascular complications occurred more often in patients undergoing BEV implantations. This may be attributed to the fact that the NAVITOR device is delivered sheathless with a 14F, flexible delivery system [11,25,26].

While pre-dilatation is a commonly used practice in NAVITOR procedures, post-dilatation is optional. Our data indicate that implantations in small annuli lead to an increase in post-dilatations not only in the SEV device but also in the BEV system. These results may reflect both an overall increased rate of not fully expanded prothesis after initial release and an attempt to compensate for generally unfavorable hemodynamics in this particularly challenging anatomy regardless of the chosen platform.

As mentioned above, 30-day mortality was not statistically different between the groups. However, numerically, a higher number of deaths occurred in the NAVITOR group. Despite the fact that the overall study population was small, making any interpretation difficult, NAVITOR patients were older and suffered more often from renal insufficiency pre-operatively, indicating that this group—reflected by the higher EuroScore—may have been more fragile, and thus prone to complications. There was no common denominator linking these cases.

### Limitations

The present analysis included a rather small number of patients not allowing for a meaningful multivariate or matched-pairs analysis and is further limited by its retrospective, non-randomized nature. However, our data on this particular cohort of patients may provide a glimpse of the results of future randomized trials. There was no adverse event monitoring, and imaging data were not analyzed by a core laboratory. The lack of long-term follow-up limits the assessment of PPMs, particularly as a predictor of long-term outcome.

## 5. Conclusions

The NAVITOR system may offer a favorable hemodynamic profile compared to current Sapien 3 Ultra device in patients with small aortic annuli. Pacemaker implantation rates remain elevated. Precise implantation depths may become easier with the recently introduced design of the next NAVITOR generation. However, future trials will have to show the actual impact of current modifications such as the new Resilia technology of the Sapien platform with regard to hemodynamic performance and valve deterioration.

## Figures and Tables

**Table 1:** Baseline characteristics.

Variable	Navitor	Ultra	*p* Value
	n = 104	n = 75	
Age, years	83.0 [79.0; 87.0]	80.0 [72.5; 84.5]	0.001
Female gender, %	91 (87.5%)	63 (84.0%)	0.654
BMI, kg/m^2^	26.7 [23.6; 29.7]	26.2 [23.9; 28.8]	0.780
NYHA IV	3 (2.9%)	3 (4.0%)	0.696
EuroSCORE II, %	3.8 [2.1; 5.0]	2.7 [2.0; 4.3]	0.072
eGFR, ml/min/1.73 m^2^	52.5 [35.0; 65.2]	62.0 [46.5; 72.0]	0.006
Peripheral artery disease	22 (21.2%)	15 (20.0%)	0.999
Prior stroke	11 (10.6%)	8 (10.7%)	1.000
Atrial fibrillation	38 (36.5%)	24 (32.0%)	0.638
Coronary artery disease	74 (71.2%)	49 (65.3%)	0.506
Prior coronary intervention	32 (30.8%)	27 (36.0%)	0.566
Echocardiographic data			
*Left ventricular ejection fraction, %*	61.0 [55.0; 64.2]	57.0 [55.0; 64.0]	0.141
*Mean aortic valve gradient, mmHg*	40.0 [30.0; 49.0]	43.0 [38.0; 51.8]	0.030
*EOA, cm^2^*	0.7 [0.6; 0.8]	0.7 [0.6; 0.9]	0.980
Electrocardiographic data			
Right bundle branch block	3 (2.9%)	2 (2.7%)	1.000
Left bundle branch block	2 (1.9%)	2 (2.7%)	1.000
Atrioventricular block	13 (12.5%)	7 (9.6%)	0.718
MDCT data			
Annulus Area (mm^2^)	392.5 [372.8; 411.3]	400.0 [373.0; 416.5]	0.267
Annulus diameter, mm	22.8 [22.1; 23.2]	22.9 [22.1; 23.3]	0.392
Aortic valve calcification, AU	1615.0 [974.0; 2319.0]	2083.0 [1194.5; 2930.0]	0.010
Calcium density (AU/cm^2^)	416.8 [272.4; 586.1]	513.3 [318.3; 742.0]	0.013

Abbreviations: BMI = body mass index; eGFR = estimated glomerular filtration rate; EOA = estimated (aortic valve) orifice area.

**Table 2:** Procedural outcomes and complications.

Variable	Navitor	Ultra	*p* Value
	n = 104	n = 75	
Procedural parameter			
Prosthesis size			<0.001
23	8 (7.7%)	68 (90.7%)	
25	57 (54.8%)	0 (0.0%)	
26	0 (0.0%)	7 (9.3%)	
27	39 (37.5%)	0 (0.0%)	
Procedural duration, min	46.5 [40.0; 60.0]	43.0 [40.0; 55.0]	0.073
Contrast agent, ml	130.0 [100.8; 155.2]	100.0 [90.0; 120.0]	<0.001
Pre-dilatation, %	100 (96.2%)	5 (6.7%)	<0.001
Post-dilatation, %	31 (29.8%)	8 (10.7%)	0.004
Echocardiographic outcome			
Left ventricular ejection fraction, %	60.0 [55.0; 64.2]	60.0 [55.0; 65.0]	0.980
Mean aortic valve gradient, mmHg	8.0 [6.0; 10.0]	13.0 [10.0; 16.0]	<0.001
EOA, cm^2^	1.9 [1.7; 2.2]	1.7 [1.4; 2.0]	<0.001
iEOA, cm^2^/m^2^	1.1 [0.9; 1.2]	1.0 [0.8; 1.1]	<0.001
Severe PPM	0 (0.0%)	2 (3.2%)	0.152
Moderate-Severe PPM	12 (12.2%)	21 (33.3%)	0.002
Relevant PVL (>mild/trace or VinV)	1 (1.0%)	1 (1.3%)	1.000
Elevated Gradients (>19 mmHg)	0 (0.0%)	6 (8.2%)	0.005
Clinical outcome			
Technical success	102 (98.1%)	70 (93.3%)	0.132
Device success at 30 days	98 (94.2%)	60 (80.0%)	0.007
30 day mortality	4 (3.8%)	1 (1.3%)	0.401
Conversion to sternotomy	0 (0.0%)	0 (0.0%)	1.000
Multiple valves (VinV)	1 (1.0%)	0 (0.0%)	1.000
Device migration/embolization	2 (1.9%)	0 (0.0%)	0.510
Major vascular complication	0 (0.0%)	5 (6.7%)	0.012
Bleeding (type 3–4)	6 (5.8%)	5 (6.7%)	1.000
Major cardiac structural complication	1 (1.0%)	2 (2.7%)	0.573
Disabling Stroke	3 (2.9%)	1 (1.3%)	0.641
AKI (type 2–4)	2 (1.9%)	0 (0.0%)	0.510
New permanent pacemaker ^1^	18 (18.4%)	6 (8.5%)	0.110

Abbreviation: EOA = estimated (aortic valve) orifice area; iEOA= indexed estimated orifice area; PVL = paravalvular leak; CNS = central nervous system; VinV = valve-in-valve; PPM = prosthesis-patient mismatch; AKI = acute kidney injury. ^1^ Excluded patients with pacemakers at baseline.

## Data Availability

Data is contained within the article.

## Abbreviations

BEV	balloon-expanding valve
iAVA	indexed aortic valve area
IAV	intra-annular valve
MDCT	multidetector computed tomography
PVL	paravalvular leakage
SAV	supra-annular valve
SEV	self-expanding valve
TAVR	transcatheter aortic valve replacement
THV	transcatheter heart valve
PPM	prosthesis-patient mismatch
